# Effect of priming injections of luteinizing hormone-releasing hormone on spermiation and ovulation in Gϋnther's Toadlet, *Pseudophryne guentheri*

**DOI:** 10.1186/1477-7827-9-68

**Published:** 2011-05-20

**Authors:** Aimee J Silla

**Affiliations:** 1 School of Animal Biology, The University of Western Australia, Perth, Australia

## Abstract

**Background:**

In the majority of vertebrates, gametogenesis and gamete-release depend on the pulsatile secretion of luteinizing hormone-releasing hormone (LHRH) from the hypothalamus. Studies attempting to artificially stimulate ovulation and spermiation may benefit from mimicking the naturally episodic secretion of LHRH by administering priming injections of a synthetic analogue (LHRHa). This study investigated the impact of low-dose priming injections of LHRHa on gamete-release in the Australian toadlet *Pseudophryne guentheri*.

**Methods:**

Toadlets were administered a single dose of two micrograms per. gram LHRHa without a priming injection (no priming), or preceded by one (one priming) or two (two priming) injections of 0.4 micrograms per. gram LHRHa. Spermiation responses were evaluated at 3, 7 and 12 hrs post hormone administration (PA), and sperm number and viability were quantified using fluorescent microscopy. Oocyte yields were evaluated by stripping females at 10-11 hrs PA. A sub-sample of twenty eggs per female was then fertilised (with sperm obtained from testis macerates) and fertilisation success determined.

**Results:**

No priming induced the release of the highest number of spermatozoa, with a step-wise decrease in the number of spermatozoa released in the one and two priming treatments respectively. Peak sperm-release occurred at 12 hrs PA for all priming treatments and there was no significant difference in sperm viability. Females in the control treatment failed to release oocytes, while those administered an ovulatory dose without priming exhibited a poor ovulatory response. The remaining two priming treatments (one and two priming) successfully induced 100% of females to expel an entire clutch. Oocytes obtained from the no, or two priming treatments all failed to fertilise, however oocytes obtained from the one priming treatment displayed an average fertilisation success of 97%.

**Conclusion:**

Spermiation was most effectively induced in male *P. guentheri *by administering a single injection of LHRHa without priming. In contrast, female *P. guentheri *failed to ovulate without priming. A single priming injection induced the release of oocytes of high viability compared to oocytes obtained from females in the two priming treatment which underwent a process of over-ripening.

## Background

Assisted Reproductive Technologies (ART), including the hormonal induction of sperm-release (spermiation), oocyte-release (ovulation) and *in-vitro *fertilisation, have enormous potential to assist the propagation of the worlds declining amphibian species [1,2]. The reliable and efficient collection of fresh gametes (spermatozoa and oocytes) is a pivotal component of ART, and for the purpose of species conservation, should be attempted via non-invasive protocols that ensure the survival of genetically viable individuals. The exogenous hormones human chorionic gonadotropin (hCG) and luteinizing hormone-releasing hormone (LHRH) have been used to induce spermiation [3-10] and ovulation [11-15] in live anurans for more than 70 years. The earliest anuran spermiation and ovulation induction protocols were developed as human pregnancy tests, whereby detoxicated urine was injected into an adult frog or toad and gamete-release monitored [3,11]. The bioactive constituent of human pregnancy urine that induced gamete-release in test animals was hCG [16]. Despite the historical use of hCG to successfully induce gamete release in a small number of model species, the use of synthetic LHRHa is largely replacing hCG as a generally more effective and reliable alternative.

The decapeptide LHRH (also called gonadotropin-releasing hormone, or GnRH), produced by the hypothalamus, is directly responsible for the synthesis and release of luteinizing hormone (LH) and follicle-stimulating hormone (FSH) from the anterior pituitary [17]. Once released, circulating LH and FSH bind to target receptors on the gonads, which respond by secreting sex steroids (such as estrogen, progesterone and testosterone) and inducing oogenesis and ovulation in adult females, and spermatogenesis and spermiation in adult males [17]. For the majority of vertebrates, normal reproductive function is dependant on the pulsatile secretion of LHRH, which precedes the release of discrete pulses of LH and FSH [17]. The pulsatile nature of LHRH release is important because it allows the pituitary gonadotropes to be 'primed' and sensitized to further stimulation [18]. Studies attempting to artificially stimulate ovulation and spermiation may therefore benefit from mimicking the naturally episodic secretion of LHRH via the use of priming injections.

Pulsatile administration of LHRH has proven to be effective at stimulating ovulation in a variety of animals including ungulates [19-22], marsupial mammals [23,24], higher primates [25], and humans [26-28]. A study by Rodger & Mate [23] on the Australian Brush-tailed Possum (*Trichosurus vulpecular*) found the administration of a single injection of synthetic LHRH (3 days after the injection of pregnant mares' serum gonadotrophin, PMSG) to be ineffective at stimulating ovulation. In contrast, administration of three low-dose injections of LHRHa (spaced 90 mins apart, 3 days after the injection PMSG) induced ovulation in a predictable and reliable manner [23]. Similarly, the most effective protocol found to stimulate ovulation in the Tammar Wallaby (*Macropus eugenii*) was three low-dose LHRH injections administered at 3 hr intervals (2-3 days after PMSG)[24]. In amphibians the natural pulsatile release of LHRH is yet to be quantified *in vivo *[17]. Despite this, a recent study by Browne *et al*. [13] tested the effectiveness of priming injections (administered at 3-4 day intervals prior to a higher 'ovulatory' dose) to induce ovulation in the endangered Wyoming Toad (*Bufo baxteri*). The administration of two low-dose priming injections stimulated the release of a significantly greater number of oocytes with improved survivorship compared to those released following no priming, or a single priming injection [13]. Whether the use of priming injections similarly improves the ovulation response of other anuran species remains to be determined.

Hormonal priming may also enhance the efficacy of spermiation induction protocols. Most previous attempts to induce spermiation in anurans have relied on a single injection of LHRH [8-10,29], but there is limited evidence to suggest that multiple injections can improve LH-release *in vivo*. A study on the American Bullfrog (*Rana catesbeiana*) reported that the administration of a second low-dose (0.4 μg) injection of LHRHa potentiated the release of LH following the initial injection of equal dose [30]. In contrast, two LHRHa injections of moderate dose (2 μg) desensitised the pituitary gland, while the administration of two high doses (10 μg) of LHRHa appeared to render the pituitary completely refractory to the second injection [30]. These findings suggest that multiple injections of LHRHa prime the pituitary to further stimulation when administered at low doses. Whether this increase in circulating LH corresponds to an increase in the number or viability of sperm released is yet to be quantified *in vivo*.

To further investigate the impact of low-dose priming injections on gamete-release, this study used the Western Australian anuran *Pseudophryne guentheri*, to: 1) evaluate the effect of no, one and two priming injections on the number of spermiating males, sperm count, sperm viability and timing of sperm release; and 2) evaluate the effect of no, one and two priming injections on the number of ovulating females, oocyte yield and fertilisation success.

## Methods

The protocols described in this manuscript were performed following evaluation and approval by the University of Western Australia's Animal Ethics Committee (approval number RA/3/100/641 and RA/3/100/836).

### Study species

*Pseudophryne guentheri *is a small (26-33 mm, snout-vent length) terrestrial toadlet in the family Myobatrachidae. *P. guentheri *is widely distributed throughout temperate forests and grasslands of south-western Australia. Breeding activity commences in autumn following heavy rainfall and continues until early winter. Male toadlets excavate terrestrial burrows in moist soil along seasonally inundated drainage lines, swamps and depressions. Advertisement calls released by males attract females to the nest site, where courtship occurs. During mating, the female deposits 80-410 (mean = 224 ± 12 oocytes, n = 40, Silla unpublished data) large, singly laid eggs which undergo intracapsular embryonic development. Terrestrial embryonic development is suspended in its early stages and larval development is later initiated when tadpoles hatch in response to the flooding of the nest site following winter rainfall. This reproductive mode (terrestrial embryonic development, aquatic larvae) is shared with all species in the genus *Pseudophryne *[31].

### Animal collection and housing

The study animals were collected from a natural population located at Lake Pinjar, approximately 40 km north of Perth, Western Australia. Male toadlets used for spermiation experiments were collected between 18:00 and 24:00 hrs from May 23-25 2009. Individual males were collected by tracking their vocalisations, locating the terrestrial nest and removing the resident animal by hand. The male toadlets captured were observed broadcasting advertisement calls and exhibited pigmented vocal sacs. Female toadlets used for ovulation experiments were collected from May 13-28 2008. Female *P. guentheri *were captured in pit-fall traps positioned within the breeding chorus. Females displaying distended abdomens were considered gravid (containing mature oocytes) and were subsequently retained for use in this study.

Toadlets were transported to the laboratory where they were housed individually in plastic aquaria (220 mm L × 140 mm W × 160 mm H) containing a layer of moist sponge beneath a 10-12 cm deep soil layer, provided to allow burrowing. Toadlets were held in an artificially illuminated constant temperature room set to a 17°C day/12°C night temperature cycle and a 10.5/13.5 hr light/dark phase.

### Experiment one: Hormonal induction of spermiation

The purpose of this experiment was to compare the spermiation response of toadlets administered a single spermiation dose of 2 μg/gram body weight LHRHa (Leuprorelin acetate; Lucrin^®^) (no priming) to those receiving a priming dose of 0.4 μg/gram body weight LHRHa one hr prior to the administration of the spermiation dose (one priming), or two priming injections of 0.4 μg/gram body weight LHRHa at one and two hrs prior to the administration of the spermiation dose (two priming)(Table 1). A spermiation dose of 2 μg/g was selected as this has previously been identified as the optimal dose to induce sperm-release in this species [10]. Toadlets were randomly allocated to treatment groups (n = 8 per treatment) and there were no significant differences (ANOVA: F_3, 31 _= 0.964, *p *= 0.424) in the weight of animals in each treatment (n = 32, mean mass (g) = 2.96 ± 0.07).

**Table 1:** Hormone injection protocol used to induce spermiation

Treatment	Dose administered at 0 hrs	Dose administered at 1 hr	Dose administered at 2 hrs
**Control**	-	-	0 μg/g LHRHa
**No priming**	-	-	2 μg/g LHRHa
**One priming**	-	0.4 μg/g LHRHa	2 μg/g LHRHa
**Two priming**	0.4 μg/g LHRHa	0.4 μg/g LHRHa	2 μg/g LHRHa

Hormone dose administered and timing of injections are shown for each priming treatment (n = 8/treatment).

Hormones were diluted in 100 μL of Simplified Amphibian Ringer (113 mM NaCl, 2 mM KCl, 1.35 mM CaCl_2_, 1.2 mM NaHCO_3_) and administered to *P. guentheri *via subcutaneous injection into the dorsal lymph sac. A control treatment consisted of toadlets administered 100 μL of Simplified Amphibian Ringer (SAR), which is the vehicle for hormone administration (Table 1). Following hormone administration toadlets were placed in holding tanks (50 mm D × 90 mm H) containing three layers of sponge (20 mm W × 20 mm L × 3 mm H) moistened with distilled water. Hydrating each toadlet using this technique ensured that animals could be stimulated to urinate at each sampling period.

### Collection and assessment of spermic urine

Spermic urine was collected by gently inserting the end of a glass microcapillary tube (fire polished and cooled) into the cloaca to stimulate urination. Spermic urine was sampled at 3, 7 & 12 hrs (± 10 mins) post hormone administration. Once collected, spermic urine volume was measured by placing the microcapillary tube (50 μL, 100 mm) alongside a ruler and dividing the length by two, providing the urine volume in microlitres. To assess sperm viability, each spermic urine sample was homogenized with 5 μL of a 1:50 dilution of the nucleic acid stain SYBR-14 (Invitrogen L-7011) and incubated in the dark for 7 mins. A 2 μL aliquot of Propidium iodide was then added and the solution was incubated in the dark for a further 7 mins. A wet mount was prepared and proportion of viable sperm evaluated under a fluorescent microscope at ×20 magnification and a wavelength of 490 nm. Spermatozoa fluorescing bright green were considered viable, while those exhibiting red fluorescence were considered non-viable. The total sperm count and proportion of viable sperm per sample was determined by assessing the sperm present in each urine sample in its entirety.

### Experiment two: Hormonal induction of ovulation

Thirty-two female *P. guentheri *were randomly assigned to one of four treatment groups (n = 8 per group), with no significant differences (ANOVA: F_3, 31 _= 1.141, *p *= 0.350) in the weight of females between treatments (n = 32, mean mass (g) = 4.55 ± 0.14). The first treatment group received a priming dose of 0.4 μg/gram body weight LHRHa (Leuprorelin acetate; Lucrin^®^) at 20:00 hrs on day 0. Twenty-four hrs later females in this treatment group received a further priming dose of 0.4 μg/gram body weight LHRHa, followed by a final ovulatory dose of 2 μg/gram body weight LHRHa at 22:00 hrs on day 3 (two priming treatment) (Table 2). The 'one priming' treatment received a single priming dose of 0.4 μg.gram bodyweight^-1 ^LHRHa, followed by a final ovulatory dose of 2 μg/gram body weight LHRHa 26 hrs later. The third treatment group received only a single dose of 2 μg/gram body weight LHRHa. Finally, a control group consisted of 8 females injected with 100 μL of SAR (Table 2). All hormone doses were diluted in 100 μL of SAR and administered via subcutaneous injection into the dorsal lymph sac. Following hormone administration females were returned to their original aquaria.

**Table 2:** Hormone injection protocol used to induce ovulation

Treatment	Dose administered at 0 hrs	Dose administered at 24 hrs	Dose administered at 50 hrs
**Control**	-	-	0 μg/g LHRHa
**No priming**	-	-	2 μg/g LHRHa
**One priming**	-	0.4 μg/g LHRHa	2 μg/g LHRHa
**Two priming**	0.4 μg/g LHRHa	0.4 μg/g LHRHa	2 μg/g LHRHa

Hormone dose administered and timing of injections are shown for each priming treatment (n = 8/treatment).

### Collection of oocytes and *in-vitro *fertilisation

Females from all treatments were removed from their aquaria 10-11 hrs post administration of the ovulatory dose and the expulsion of eggs from the oviduct was facilitated by holding the frog with legs unrestrained, and gently applying pressure to the abdomen in a craniocaudal direction (a technique referred to as stripping)[32]. Females that had ovulated expelled oocytes within 90 sec of abdominal pressure being applied. Those females not expelling oocytes at this time were returned to their aquaria and stripping was reattempted every 6-12 hrs for a period of 4 days; females that did not expel their oocytes at 10-11 hrs post hormone administration did not expel their oocytes during the subsequent stripping attempts.

Oocytes from each female were collected in a dry Petri dish and oocyte yield determined. Females were weighed immediately prior to and post stripping and the average egg mass per female was determined as delta mass/oocyte yield. A subsample of twenty eggs per female were transferred to individual square trays (45 mm W × 45 mm L × 8 mm H) and fertilised using sperm from previously prepared testis macerates obtained from field caught males. To produce macerates males were killed by double-pithing and both testes were thoroughly crushed in 250-600 μL of SAR in eppendorf tubes and refrigerated at 5°C. The sperm concentration in each suspension was measured from a homogenized sub-sample using an Improved Neubauer Haemocytometer. Sperm suspensions were refrigerated for approximately 14 hrs before an aliquot was activated in 1:4 SAR to yield a 200 μL solution with a fixed concentration of 2500 sperm/μL. The sperm solution was pipetted directly onto the oocytes and the tray was agitated for 1 min. Each tray was enclosed within a Petri-dish and left to develop at room temperature (approx. 10-22°C). Developing embryos were supplied with 100 μL of deionised water at 30 mins, and a further 100 μL every 12-24 hrs post fertilisation as required to maintain adequate hydration. Fertilisation success was determined as the percentage of embryos developing to neurulation, Gosner stages 14-16 [33].

### Statistical Analyses

The number of spermiating and ovulating toadlets were compared between LHRHa priming treatments (no, one or two priming), and between each priming treatment and the control, using two-tailed Fisher's exact tests. Brown-Forsythe tests were conducted on all variables to determine homogeneity of variances prior to all other analyses. Comparison of the number of spermatozoa expelled over the 12 hr sampling period, and at individual sampling times (3, 7 and 12 hrs PA) were analysed using Welch's ANOVAs due to unequal variances. Regression analyses were conducted to determine whether the total number of spermatozoa expelled could be predicted by the volume of urine expelled, or toadlet mass. Sperm viability data was arcsine transformed using the transformation sin^-1^(√x). Transformed sperm viability and untransformed oocyte yield, oocyte mass and fertilisation success were compared between priming treatments using one-way analysis of variance (ANOVA) and Tukey-Kramer Honestly Significant Difference (HSD) *post hoc *tests. All statistical analyses were performed using the JMP 8.0.2 software package (SAS Institute Inc. 2009). For all tests in this study, *P < 0.05 *was considered significant.

## Results

### Experiment one: Hormonal induction of spermiation

Urine samples were collected from all individuals at each sampling time (3, 7 & 12 hrs) post administration (PA), with a mean volume of 25.97 ± 2.65 μL per sample. The majority of samples obtained from control animals were aspermic, however 25% (2/8) of individuals within this treatment released a small total number of spermatozoa (< 17) over the sampling period. Of the males administered no, one or two priming injections of LHRHa, 100% (8/8) of samples contained spermatozoa within three hrs of hormone treatment and continued to contain spermatozoa at 12 hrs PA. The number of spermiating males was significantly higher in priming treatments (8/8) relative to the control treatment (2/8; two-tailed Fisher Exact Tests, *p *= 0.007), but the number of spermiating males administered no, one or two priming injections of LHRHa (8/8) did not differ significantly from one another (two-tailed Fisher Exact Tests, *p *= 1.00).

**Figure 1:**
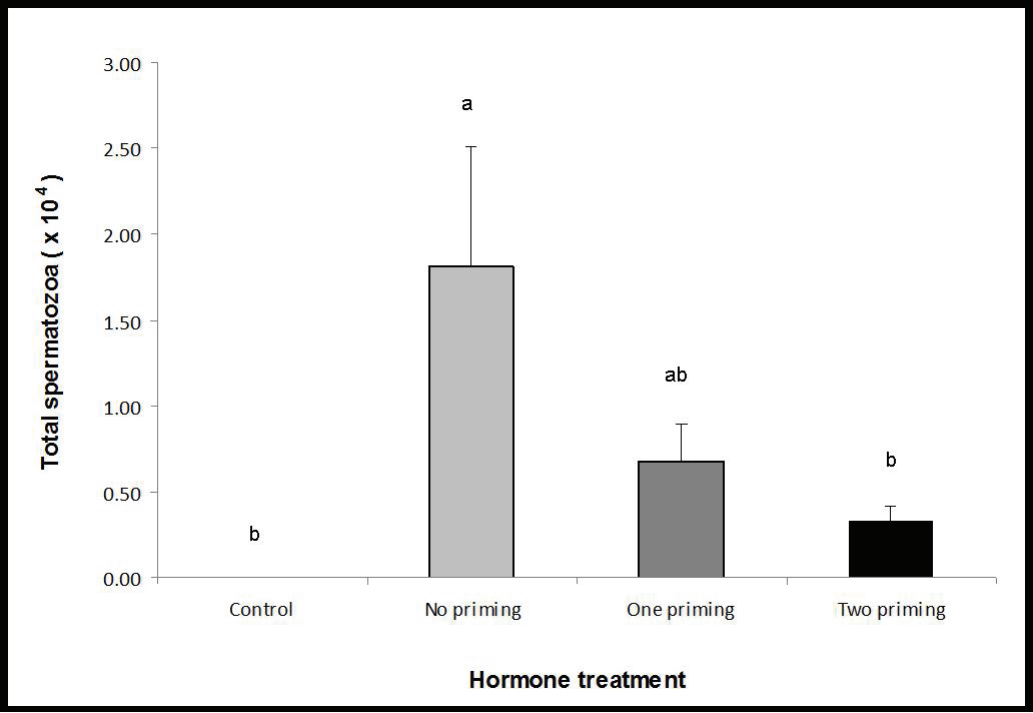
**The total number of spermatozoa (mean ± SEM) released by frogs over a 12 hr period in response to administration of control, no, one or two priming injections (n = 8/treatment)**. Data shown are mean ± SEM. Letters displayed are the result of a Tukey Kramer HSD post-hoc test, treatments that share a letter are not significantly different from each other.

The total number of spermatozoa expelled over the 12 hr sampling period differed significantly according to priming treatment (Welch's ANOVA, F_3, 11.667 _= 9.255, *p *= 0.002; Figure 1). The no priming treatment produced a significantly higher number of spermatozoa compared to the control and two priming treatments (Tukey-Kramer HSD, *P *< 0.05; Figure 1), but was not significantly higher than the one priming treatment due to high variance in the number of spermatozoa expelled (Tukey-Kramer HSD, *P *> 0.05; Figure 1). In addition, significant treatment effects were detected at each of the individual sampling times, 3 hrs (Welch's ANOVA, F_3, 11.667 _= 5.538, *p *= 0.013), 7 hrs (Welch's ANOVA, F_3, 11.667 _= 4.707, *p *= 0.022) and 12 hrs (Welch's ANOVA, F_3, 11.667 _= 5.270, *p *= 0.016) PA. The number of spermatozoa expelled by males in the no priming treatment was consistently higher than the remaining treatments at all sampling periods PA (Table 3). Peak sperm-release occurred at 12 hrs PA for all priming treatments (Table 3). The total number of spermatozoa expelled was not predicted by the volume of urine collected or toadlet mass (r^2 ^< 0.001, *p *= 0.956; r^2 ^= 0.007, *p *= 0.645, respectively).

**Table 3:** The number of spermatozoa released (× 10^3^) and sperm viability of samples collected at 3, 7 and 12 hrs post LHRHa administration

	3 hrs PA		7 hrs PA		12 hrs PA	
**Treatment**	**Sperm Count (× 10^3^)**	**Sperm Viability**	**Sperm Count (× 10^3^)**	**Sperm Viability**	**Sperm Count (× 10^3^)**	**Sperm Viability**
**Control**	0.002 ± 0.002	-	0.0003 ± 0.0003	-	0.001 ± 0.001	-
**No priming**	4.157 ± 1.754	0.558 ± 0.058	5.289 ± 2.826	0.511 ± 0.093	8.609 ± 3.153	0.540 ± 0.069
**One priming**	2.144 ± 1.061	0.703 ± 0.074	1.762 ± 0.854	0.573 ± 0.125	2.807 ± 1.159	0.653 ± 0.060
**Two priming**	0.525 ± 0.176	0.595 ± 0.104	0.962 ± 0.341	0.530 ± 0.079	1.813 ± 0.874	0.570 ± 0.064

Sperm viability was calculated as live sperm/total. n = 8/treatment. Data shown are means ± SEM.

The proportion of viable sperm (sperm viability) was calculated for all samples where a sperm count of ≥ 30 spermatozoa was achieved. The overall mean sperm viability of spermatozoa collected from males administered no, one or two priming injections of LHRHa (0.538 ± 0.057; 0.644 ± 0.048; 0.568 ± 0.036, respectively) did not differ significantly from one another (one-way ANOVA, F_2,23 _= 1.348, *p *= 0.281). Similarly, the sperm viability of spermatozoa collected at each of the individual sampling times (3, 7, and 12 hrs PA) did not differ significantly (one-way ANOVAs, p > 0.05), despite the one priming treatment inducing the expulsion of spermatozoa of consistently higher viability. The temporal effect of sampling period (3, 7 & 12 hrs) on sperm viability affected all priming treatments consistently. Maximal sperm viability occurred at 3 hrs PA, with an 8.42-18.49% drop in sperm viability at 7 hrs PA, followed by a 5.19-11.38% rise in sperm viability at 12 hrs PA (Table 3).

### Experiment two: Hormonal induction of ovulation

Oocytes could not be stripped from all females administered a control injection of SAR, while females administered an ovulatory dose of LHRHa with no priming injection, exhibited a poor ovulatory response (2/8). The remaining two priming treatments (one and two priming) successfully induced 100% of females (8/8) to expel oocytes when stripped at 10-11 hrs PA and this number was significantly higher than the control and no priming treatments (two-tailed Fisher Exact Tests, *p *< 0.05; Table 4).

**Table 4:** Comparison of the number of ovulating females administered no, one or two priming injections of LHRHa (*n *= ovulating females/females within treatment group).

	Control (*n *= 0/8)	No priming (*n *= 2/8)	One priming (*n *= 8/8)	Two priming (*n *= 8/8)
**Control**(*n *= 0/8)		0.467	0.0002*	0.0002*

**No priming**(*n *= 2/8)	0.467		0.007*	0.007*

**One priming**(*n *= 8/8)	0.0002*	0.007*		1.000

**Two priming**(*n *= 8/8)	0.0002*	0.007*	1.000	

Data shown are *P *values generated from two-tailed Fisher Exact Tests. * denotes statistical significance (P < 0.05).

The total number of oocytes expelled (oocyte yield) differed significantly according to priming treatment (one-way ANOVA, F_3,31 _= 52.219, *p *< 0.001). The one priming treatment resulted in the expulsion of a significantly greater number of oocytes compared to the control or no priming treatments (Tukey-Kramer HSD, *P *< 0.05; Table 5), but this was not significantly different to the number of oocytes expelled from females in the two priming treatment (Tukey-Kramer HSD, *P *> 0.05; Table 5). The mean mass (g) of oocytes obtained from females administered no, one or two priming injections of LHRHa (0.0011 ± 0.0016; 0.0059 ± 0.0012; 0.0073 ± 0.0007, respectively), differed significantly according to priming treatment (one-way ANOVA, F_2,17 _= 4.108, *p *= 0.038). The oocytes obtained from females receiving an ovulatory dose of LHRHa without priming, were significantly smaller than those obtained from females receiving two priming injections (Tukey-Kramer HSD, *P *< 0.05; Table 5), with oocytes obtained from a single priming injection of intermediate mass (Tukey-Kramer HSD, *P *> 0.05; Table 5). A random subset of twenty expelled oocytes per female were fertilised and the proportion developing to neurulation (fertilisation success) assessed. The mean fertilisation success of oocytes collected from females administered no, one or two priming injections differed significantly (one-way ANOVA, F_2,17 _= 773.83, *p *< 0.001). Oocytes collected from females in the one priming treatment exhibited high fertilisation rates ranging from 91-100%, while all oocytes obtained from females in the no and two priming treatments failed to fertilise (Table 5).

**Table 5:** The number of ovulating females, oocyte yield, oocyte mass and fertilisation success of oocytes obtained from females administered no, one or two priming injections of LHRHa (n = 8/treatment).

Treatment	No. females expelling oocytes	Oocyte yield		Oocyte mass (g)		Fertilisation Success	
**Control**	0	0.00 ± 0.00	a	-	-	-	-
**No priming**	2	18.75 ± 13.62	a	0.0011 ± 0.0016	a	0.00 ± 0.00	a
**One priming**	8	217.50 ± 12.53	b	0.0059 ± 0.0012	ab	0.97 ± 0.01	b
**Two priming**	8	220.13 ± 27.97	b	0.0073 ± 0.0007	b	0.00 ± 0.00	a

Fertilisation success was calculated as the proportion of embryos developing to neurulation. Data shown are mean ± SEM. Letters displayed are the result of Tukey Kramer HSD post-hoc tests, treatments that share a letter are not significantly different from each other.

## Discussion

Few studies of anuran ART have attempted to induce gamete-release by administering priming injections of synthetic LHRH, an administration protocol that aims to mimic the naturally episodic release patterns of this hormone from the hypothalamus. This study quantified the effect of no, one, or two priming injections of LHRHa on the number of spermiating individuals, sperm count, sperm viability and timing of sperm-release of male *P. guentheri*. Additionally, the ovulatory response of female *P. guentheri *administered no, one, or two priming injections of LHRHa was quantified by determining the number of ovulating females, oocyte yield and fertilisation success. The effects of low-dose priming injections on spermiation and ovulation are discussed separately.

### Hormonal induction of spermiation

All priming treatments illicited a spermiation response in *P. guentheri*, with 100% of males administered LHRHa releasing spermatozoa at each sampling period (3, 7 & 12 hrs). The number of spermiating males, and the sperm viability of spermic urine samples, did not differ according to the number of priming injections administered (no, one or two priming). This lack of difference in sperm viability among the three priming treatments indicates that the hormone protocol used did not stimulate the final stages of spermiogenesis, the process of elongation and transformation of spermatids into mature viable spermatozoa [34]. Hormonal priming injections may stimulate earlier stages of spermatogenesis, such as spermatogonium proliferation or the transformation of spermatocytes into spermatids [cf [34]], however this would need to be investigated with histological examination of the testis, which was beyond the scope of this study.

The number of spermatozoa expelled by males differed significantly according to priming treatment. Contrary to expectations, peak sperm-release occurred in the no priming treatment, with a decrease in the number of spermatozoa expelled in the one priming treatment, and a further decline identified in the two priming treatment. The observed decline in the number of spermatozoa expelled may be the result of LHRH-receptor desensitisation and down-regulation, whereby LHRH doses in excess of the optimal range induce a decline in the number or sensitivity of LHRH-receptors, reducing LH release, and subsequently impeding the spermiation response [35]. For example, a study by McCreery et al [30] on the American Bullfrog (*Rana catesbeiana*) reported that the administration of two LHRHa injections of moderate dose (2 μg) desensitised the pituitary gland, while administration of a second low-dose (0.4 μg) injection of LHRHa potentiated the release of LH [30]. In the present study a single injection of 2 μg/g LHRHa was administered as this dose was previously identified as the optimal spermiation dose for *P. guentheri *[10]. This injection was preceded by the administration of no, one or two priming injections of 0.4 μg/g LHRHa. It is possible that the cumulative dose of LHRHa administered (2.0, 2.4 or 2.8 μg/g) led to pituitary desensitisation, resulting in the release of a lower number of spermatozoa in the one and two priming treatments respectively.

An alternative explanation for the observed decline in spermatozoa output is an additive stress response to multiple injections, due primarily to increased handling and needle puncture. It is well established that the handling and restraint of individuals causes physiological stress in a variety of vertebrates [36-39]. The increase in plasma corticosterone associated with stress inhibits reproduction [40] by blocking the release of LHRH from the hypothalamus [41]. More specifically, the process of spermatogenesis, and the quality and quantity of spermatozoa spermiated, have been shown to decline in response to elevated circulating corticosterone concentrations [42,43]. To determine whether corticosterone levels increase in response to multiple injections, plasma samples would need to be obtained and analysed via radioimmunoassay (RIA) techniques. These techniques may have limited application in small anurans, such as *P. guentheri*, where the collection of sufficient blood volumes are restricted. An alternate approach is the analysis of urinary and faecal metabolites using high performance liquid chromatography (HPLC), though limitations in the sensitivity of this technique should also be considered [cf [44,45]]. If corticosterone levels are found to increase in response to multiple injections, alternate hormone administration protocols aimed at reducing stress, such as topical application or controlled-release implants may be implemented in order to improve the spermiation response.

The majority of published studies inducing spermiation in anurans have used a single injection of hCG or LHRHa to promote sperm-release [6,8-10,14,15]. Results from this study confirm that a single injection of LHRHa is more effective than multiple priming injections at stimulating spermiation in the terrestrial toadlet *P. guentheri*. A single injection protocol is therefore recommended for future sperm-release induction studies in this species.

### Hormonal induction of ovulation

Data from this experiment show clear differences in the ovulatory responses of female *P. guentheri *administered no, one or two priming injections of LHRHa. An 'ovulatory' dose of LHRHa administered without priming was insufficient to stimulate ovulation in the majority of females tested, with only two of eight females in this treatment group releasing oocytes. The two females able to be stripped of oocytes released a partial clutch (mean = 18.75 ± 13.62) substantially smaller than the average clutch size of the species (mean = 224 ± 12 oocytes, n = 40, Silla unpublished data) and not statistically different from the number of oocytes obtained from control females (0.00 ± 0.00). In contrast, all females administered one or two priming injections were able to be stripped of an entire clutch (mean = 217.50 ± 12.53; 220.13 ± 27.97, respectively). These results are comparable to the observations of Browne *et al.*[13], where female *Bufo baxteri *failed to ovulate without the administration of one or two priming injections. Similarly, the protocol employed to stimulate ovulation in *Xenopus tropicalis *involves administering a single priming injection to females prior to an ovulatory dose [46]. Combining the results of the present study, with those of Browne *et al*. [13], indicate that in some anuran species priming injections are necessary to sensitise the ovary and ensure successful ovulation following the administration of the ovulatory dose.

Interestingly, oocytes obtained from females in the two priming treatment could not be fertilised, while those obtained from females in the one priming treatment exhibited consistently high fertilisation success (0.97 ± 0.01). These results contrast with the observations of Browne *et al*. [13], where oocytes collected from female *Bufo baxteri *administered two priming injections displayed a significantly greater proportion of embryos developing to neurulation than those from one priming (0.13 ± 0.03; 0.06 ± 0.04, respectively). It is important to note however, that overall the proportion of embryos developing to neurulation reported by Browne *et al*. [13] were substantially lower than those observed from the one priming treatment in the present study (see above). A plausible explanation for the diminished fertilisation capacity of oocytes obtained from females administered two priming injections in the present study, is oocyte over-ripening. Over-ripening is the process of aging of the oocytes retained within the coelomic cavity of the female post ovulation [cf [47]]. Over-ripened oocytes are commonly reported in broodfish that do not oviposit spontaneously in captivity [47] and over-ripening is always associated with a substantial decrease in egg viability [48,49]. The terrestrial toadlet *P. guentheri*, as with other terrestrial myobatrachids, does not usually oviposit spontaneously following hormonal induction of ovulation in captivity (pers obs). Instead, physical stimulation of the oviduct through the process of stripping is required to obtain oocytes in this species. It is possible that females administered two priming injections of LHRHa ovulated earlier, and subsequently retained their oocytes longer, than those administered one priming injection, despite females in all treatments being stripped at a standard 10-11 hrs PA. This would lead to the over-ripening of oocytes in the two priming treatment, as indicated by the loss of fertilisation capacity (egg viability) and increase in the wet mass of oocytes [49].

## Conclusion

The administration of priming injections of synthetic LHRH, an administration protocol that aimed to mimic the naturally episodic release patterns of this hormone from the hypothalamus, produced different results for male and female *P. guentheri*. In male *P. guentheri*, a single injection of LHRHa without priming was most effective at stimulating spermiation. In contrast, female *P. guentheri *failed to ovulate without priming, while a single priming injection induced the release of oocytes displaying high fertilisation success. The administration of a second priming injection induced the release of oocytes that had undergone a process of over-ripening and loss of fertilisation capacity.

## Competing interests

The author declares that they have no competing interests.

## Authors' contributions

AJS designed the study, performed all experimental procedures and wrote the paper.
